# New objective timbre parameters for classification of voice type and fach in professional opera singers

**DOI:** 10.1038/s41598-022-22821-w

**Published:** 2022-10-26

**Authors:** Matthias Müller, Zehui Wang, Felix Caffier, Philipp P. Caffier

**Affiliations:** 1 Department of Audiology and Phoniatrics, Charité-Universitätsmedizin Berlin, Campus Charité Mitte, Charitéplatz 1, 10117 Berlin, Germany; 2 Institute for Digital Transformation, University of Applied Sciences Ravensburg-Weingarten, Doggenriedstraße, 88250 Weingarten, Germany; 3 HTW Berlin University of Applied Sciences, Treskowallee 8, 10318 Berlin, Germany

**Keywords:** Acoustics, Biomedical engineering, Preventive medicine

## Abstract

Voice timbre is defined as sound color independent of pitch and volume, based on a broad frequency band between 2 and 4 kHz. Since there are no specific timbre parameters, previous studies have come to the very general conclusion that the center frequencies of the singer’s formants are somewhat higher in the higher voice types than in the lower ones. For specification, a database was created containing 1723 sound examples of various voice types. The energy distribution in the frequency bands of the singer’s formants was extracted for quantitative analysis. When the energy distribution function reached 50%, the corresponding absolute frequency in Hz was defined as Frequency of Half Energy (FHE). This new parameter quantifies the timbre of a singing voice as a concrete measure, independent of fundamental frequency, vowel color and volume. The database allows assigning FHE means ± SD as characteristic or comparative values for sopranos (3092 ± 284 Hz), tenors (2705 ± 221 Hz), baritones (2454 ± 206 Hz) and basses (2384 ± 164 Hz). In addition to vibrato, specific timbre parameters provide another valuable feature in vocal pedagogy for classification of voice type and fach according to the lyric or dramatic character of the voice.

## Introduction

In singing voice pedagogy, auditory perception indicates that dramatic voices sound darker than lyric ones. However, since this is only an empirical experience, an objective parameter needs to be developed in order to objectify and classify timbre. The timbre of a singing voice is traditionally defined as a characteristic sound property^1^. This definition of timbre is usually summarized as a perceptual quantity independent of pitch and loudness^2^. Individual timbre is particularly evident in the higher frequency components of the sound, the so-called singer’s formants (SF)^3,4^. In both the literature on singing and the pedagogy of singing, the term timbre is also frequently used as the individual color of a voice^5,6^. The 3rd, 4th and 5th SF (i.e., F3, F4, F5) were found to be important for the timbre and carrying power of the voice^7,8^. It is described, how a voice synthesizer can be used to simulate a higher voice type by raising these three frequencies by 200 Hz each^8^. This method can be applied to train the ability of assigning the timbre to a certain voice type. According to Sundberg, the center frequencies of the SF are somewhat higher in the higher voice types than in the lower ones^9^, with F3, F4 and F5 clustered in a wide band around 3 kHz. Following these observations, it becomes clear that the position of the SF is important in assigning a sound to a voice type. If these frequency bands are higher, voices are perceived as brighter and assigned to a higher voice type; if they are lower, they are perceived as darker and assigned to a lower one. For the voice types tenor, baritone, and bass, the frequently used terms *tenor timbre*, *baritone timbre*, and *bass timbre* represent bright, darker, and very dark sounding voices. The related frequency bands are based on individual measurements or on model calculations^1,10^.

In order to objectively discuss the issue of voice classification within singing voice pedagogy, various classification systems must be clearly distinguished^11^. “Voice type” is merely a basic term describing the pitch range in which different voices sing. Usually, the different voice types are summarized in two groups. In the higher voices, the soprano, mezzo, and alto voice types are distinguished from the tenor, baritone, and bass voice types in the lower voices, respectively from high to low. Traditionally, female parts of the various operas are cast with voice types from the first group, and male parts with the voice types from the group with the lower voices. The few exceptions, such as the breeches roles (or “Hosenrollen”), in which a male part is cast with a high voice, will not be considered in particular here. For stage practice in opera singing, voices are also classified according to specific qualities and singing requirements. In these classifications, such as the traditional German “Fach” system, voices can be categorized as either lyric or dramatic^12,13^. Following Ling^13^, we use the term “voice structure” for this distinction. The differentiation of lyric vs. dramatic is often essential to professional opera singers because of the different demands on vocal performance^14^. Dramatic voices require great carrying power and projection, because the orchestral accompaniment for dramatic roles is often characterized by high sound pressure levels. To meet these demands, the singer must be able to sustain high volume levels for long periods of time. In contrast, a lyric role often requires a high degree of vocal agility and a particular overall sound aesthetic. Misclassification of voice type and voice structure can lead to overuse and result in both functional and organic dysphonia. This usually requires intensive logopedic therapy and even invasive phonosurgical treatments in addition to vocal pedagogical and phoniatric consultation^15–18^.

In our research project, a database was needed to classify professional opera voices into a lyric or dramatic category within their voice types. The overall goal is to find suitable objective characteristics for voice classification. Recently, vibrato was found to be a very good feature for classifying voices as lyric or dramatic^19^. The purpose of this study is to investigate whether timbre parameters can also play a role in this classification. These parameters should be able to describe the color of a voice as a numerical quantity, independent of pitch, loudness, and vowel color. Independence from the latter is particularly important, since professional opera singers can use vowel color to deliberately shape any sound given by the phonetics of the language, either bright or dark. Spectral analysis shows that this is achieved by either raising or lowering the 2nd vowel formant (F2) through vocal tract adjustments^20,21^. Auditory differentiation of vowel color from the overall voice timbre is essential in singing voice pedagogy and requires professional experience. The new timbre parameters should not only allow an objective comparison of sound color between different voice types, but also between the lyric and dramatic voice structure within each voice type.

## Materials and methods

At the Institute for System Theory and Signal Processing at the University of Stuttgart, a system was developed for classifying professional soprano, tenor, baritone and bass opera voices. To realize this system, various features were developed in a Matlab environment in order to analyze sound samples from lyric and dramatic opera singers within each voice type.

### Database

A comprehensive database with a total of 1723 sound examples, all of which can be clearly assigned to a lyric or dramatic category, was compiled and implemented in a Matlab environment. For each sound sample, an analysis interval was defined, containing a single tone with a particular, consistent vowel color. To preserve the original musical context of the investigated tone, every sound sample in the database had a duration of up to 12 s. Furthermore, the samples were labeled with codes containing the following information: voice type, voice structure (lyric or dramatic), performer name, pitch, vowel, and title of the musical work. From the 1723 sound samples, 774 were sopranos (352 lyric, 422 dramatic), 389 tenors (199 lyric, 190 dramatic), 343 baritones (157 lyric, 186 dramatic), and 217 basses (60 lyric, 157 dramatic). A more detailed description of this extensive database can be found in a previous study^19^.

### Development and calculation of objective timbre parameters

The parameters for objectifying and specifying the timbre should be able to describe characteristic properties of the spectrum, independent of vowel color. Consequently, a spectral centroid above the vowel formats had to be found. Based on the knowledge of singing voice acoustics, especially the position of vowel formants, as well as the analysis of the extensive sound samples of our database, the following frequency bands $\left[{B}_{1},{B}_{2}\right]$ were determined:

- soprano 2300–4500 Hz
- tenor 2000–3600 Hz
- baritone 2000–3600 Hz
- bass2000–3600 Hz

For all frequency bands, the center of the sound energy was calculated. To ensure comparability between both voice types and voice structure, the cutoff frequencies were defined to be the same within each gender.

Since different voice types and voice categories produce different sound spectra, this can be used to develop a possible classification. In this study, specific features based on distribution of energy in the SF frequency bands were extracted for quantitative analysis. The energy distribution function $P(f)$ is defined as

1 $$P(f)=\frac{\sum_{i={B}_{1}}^{f}{S}^{2}(i)}{\sum_{i={B}_{1}}^{{B}_{2}}{S}^{2}(i)}$$

where $\left[{B}_{1},{B}_{2}\right]$ is the frequency interval corresponding to each voice type.

When the energy distribution function $P(f)$ reaches 50% of the whole, the corresponding absolute frequency is defined as Frequency of Half Energy (FHE in Hz), i.e. $P\left({f}_{FHE}\right)=0.5$. The relative position in the frequency interval is defined as Position of Half Energy (PHE in %), which is calculated as

2 $$PHE=\frac{{f}_{FHE} -{B}_{1}}{{B}_{2} - {B}_{1}}*100\%$$

However, PHE or FHE can only represent the midpoint position of the energy distribution and cannot extract further information from the entire SF frequency range. Therefore, this paper defines the energy density function as

3 $$p(f)=\frac{{S}^{2}(f)}{\sum_{i={B}_{1}}^{{B}_{2}}{S}^{2}(i)}$$

Since the moments of a function are quantitative measures related to the shape of the function's graph, the first-order moments of the energy density function are defined as the Spectral Centroid (SC), which is calculated as

4 $$Centroid(f)=\sum_{f={B}_{1}}^{{B}_{2}}f * p(f)$$

$Centroid(f)$ represents the estimator of the energy function in the frequency interval, i.e. the energetic center of the specified SF frequency range. For the listener, this criterion is essential for the "brightness" of the sound. Similarly, the 2nd, 3rd and 4th order moments are defined as spectral variance, skewness and kurtosis respectively, which also describe SF features.

### Statistical analysis

Basic timbre data analysis was performed using descriptive statistics by calculating means and standard deviations (SD) separately for all singers, each voice type (soprano, tenor, baritone, bass) and voice structure (lyric vs. dramatic). To compare the mean values of FHE, PHE and SC depending on voice structure, a two-tailed t-test for independent samples was applied after checking that skewness of the distribution ranged between -1 and 1 in all subgroups. Additionally, we computed reference ranges of FHE, PHE and SC (Mean ± 1.96 × SD) including voice structure-specific differences in all voice types.

Furthermore, machine learning methods were applied for voice structure classification. Random forest, an excellent method in ensemble learning, was used to significantly improve the accuracy of classification problems. This is achieved by growing an ensemble of base learners and letting them vote for the most popular class^22^. Base learners in random forest, i.e. decision trees, randomly select features to increase robustness with respect to noise^23^. To enhance the accuracy of this model, additional dimensions had to be considered. Besides timbre parameters, the following critical features were extracted for the classification procedure: vibrato parameters (rate of vibrato, VR; extent of vibrato, VE), formant characteristics (strength of formant; start and stop of formant), and perturbation measures (jitter; shimmer). The ability of the random forest model to analyze the impact of each input feature on the classification was based on information gain during the classification process.

We chose line graphs, correlation heat maps and bar charts as graphical techniques to represent the data. Moreover, the SHapley Additive exPlanations (SHAP), a tool for visualizing machine learning models, was used to explain the output of the training model. All statistical tests were done using SPSS version 26.0.0.1 (IBM, Armonk, NY). The level of significance was set at α = 0.05. The graphics were created using MATLAB R2017b (The MathWorks, Inc., Natick, MA) and Python version 3.8 (Python Software Foundation, Wilmington, DE). All classification procedures were implemented by Scikit-learn version 1.0.1, the open-source machine learning package in Python.

## Results

The mean values, SD and 95% reference rages (RR) for the newly introduced timbre parameters FHE, PHE and SC are shown in Table 1. Data are presented for all voice types and each voice structure (lyric vs. dramatic).

**Table 1:** Timbre parameters frequency of half energy (FHE), position of half energy (PHE) and spectral centroid (SC) depending on voice structure (lyric vs. dramatic) in all voice types.

Vocal characteristics of investigated opera singers		Frequency of half energy (FHE in Hz)			Position of half energy (PHE in %)			Spectral centroid (SC in Hz)		
Voice type	Voice structure (*n* sound samples)	Mean (SD)	95% RR	*p*-value (*t*-test)	Mean (SD)	95% RR	*p*-value (*t*-test)	Mean (SD)	95% RR	*p*-value (*t*-test)
Soprano	All (n = 774)	3092 (284)	3072; 3112		35.98 (12.92)	35.07; 36.89		3207 (154)	3196; 3218	
	Lyric (n = 352)	3169 (307)	3136; 3201	< 0.001	39.46 (13.94)	38.00; 40.92	< 0.001	3271 (159)	3255; 3288	< 0.001
	Dramatic (n = 422)	3028 (247)	3004; 3052		33.08 (11.23)	32.00; 34.15		3153 (128)	3141; 3166	
Tenor	all (n = 389)	2705 (221)	2683; 2727		44.05 (13.82)	42.67; 45.43		2740 (143)	2725; 2754	
	lyric (n = 199)	2760 (205)	2732; 2789	< 0.001	47.49 (12.77)	45.70; 49.28	< 0.001	2789 (129)	2771; 2807	< 0.001
	dramatic (n = 190)	2648 (224)	2616; 2680		40.45 (13.99)	38.45; 42.45		2688 (139)	2668; 2708	
Baritone	all (n = 343)	2454 (206)	2432; 2476		28.34 (12.86)	26.98; 29.71		2532 (144)	2516; 2547	
	lyric (n = 157)	2576 (206)	2543; 2608	< 0.001	35.94 (12.86)	33.91; 37.97	< 0.001	2625 (135)	2604; 2646	< 0.001
	dramatic (n = 186)	2351 (140)	2331; 2371		21.93 (8.72)	20.67; 23.20		2453 (97)	2439; 2467	
Bass	all (n = 217)	2384 (164)	2363; 2406		24.01 (10.24)	22.64; 25.38		2480 (92)	2468; 2493	
	lyric (n = 60)	2379 (200)	2327; 2430	0.749	23.65 (12.46)	20.43; 26.87	0.747	2491 (98)	2466; 2516	0.284
	dramatic (n = 157)	2387 (149)	2363; 2410		24.15 (9.30)	22.68; 25.61		2476 (89)	2462; 2490	

*n* number, *RR* reference ranges (lower bound; upper bound), *SD* standard deviation.

As expected, the FHE values were larger for the higher voice types than for the lower ones. The means revealed a difference of 251 Hz between the tenor (2705 Hz) and the baritone timbre (2454 Hz). The mean difference between baritone and bass voices (2384 Hz) was much smaller, i.e. 70 Hz. Moreover, within each voice type, a difference between the two voice categories was evident. In general, for the sopranos, tenors, and baritones, lyric voices had a significantly higher FHE mean than the dramatic voices (p < 0.001). For the baritones, this difference was the largest (225 Hz), only slightly smaller than the difference between a tenor and a baritone timbre. For the bass voices, no significant mean difference (p = 0.749) with respect to voice structure could be determined (8 Hz). Also, the smallest SD of the FHE values was found for the basses compared to other voice types. Regarding voice categories, the SD of the FHE means within the sopranos and baritones were considerably larger in the lyric than the dramatic voices, while it was the opposite for the tenors. These FHE findings were also reflected by the results of the parameters PHE and SC.

As an example for individual data, Table 2 presents the means and SD of the sound samples taken from the lyric baritone (B2) Mario Cassi, the dramatic tenor (T1) Plácido Domingo, the dramatic sopranos (S1) Birgit Nilsson and Nina Stemme, and the lyric soprano (S2) Daniela Mazzucato.

**Table 2:** Individual results of the investigated timbre and vibrato parameters for the elite vocal performers Plácido Domingo, Mario Cassi, Birgit Nilsson, Nina Stemme and Daniela Mazzucato. For easier comparison, the 95% reference ranges represent the data of all investigated singers of the corresponding voice type and voice structure (lyric vs. dramatic), taken from Table 1 (FHE, PHE, SC) and Müller et al.^17^ (VR, VE).

Parameter	Domingo (T1)	Cassi (B2)	Nilsson (S1)	Stemme (S1)	Mazzucato (S2)
**Sample size (Timbre/Vibrato)**	49/49	21/21	95/93	25/25	31/27
**FHE (in Hz)**					
Mean ± SD	**2581 ± 162**	**2886 ± 158**	**2942 ± 227**	**2931 ± 142**	**3468 ± 300**
*95% RR*	2616; 2680	2543; 2608	3004; 3052	3004; 3052	3136; 3201
**PHE (in %)**					
Mean ± SD	36.28 ± 10.09	55.33 ± 9.88	29.16 ± 10.37	28.65 ± 6.46	53.05 ± 13.63
*95% RR*	38.45; 42.45	33.91; 37.97	32.00; 34.15	32.00; 34.15	38.00; 40.92
**SC (in Hz)**					
Mean ± SD	2626 ± 82	2814 ± 83	3112 ± 109	3049 ± 81	3459 ± 146
*95% RR*	2668; 2708	2604; 2646	3141; 3166	3141; 3166	3255; 3288
**VR (in Hz)**					
Mean ± SD	**6.26 ± 0.45**	**5.84 ± 0.39**	**6.06 ± 0.51**	**6.20 ± 0.26**	**6.21 ± 0.50**
*95% RR*	5.93; 6.08	6.01; 6.21	6.10; 6.23	6.10; 6.23	6.29; 6.47
**VE (in Cent)**					
Mean ± SD	**74.05 ± 16.04**	**130.10 ± 25.73**	**74.58 ± 20.80**	**122.76 ± 29.77**	**77.77 ± 25.11**
*95% RR*	80.17; 86.29	102.38; 112.31	95.76; 102.38	95.76; 102.38	73.28; 79.32

*B2* lyric baritone, *FHE* Frequency of Half Energy, *PHE* Position of Half Energy, *RR* reference ranges (lower bound; upper bound), *S1* dramatic soprano, *S2* lyric soprano, *Sample size* number of investigated sound samples, *SC* spectral centroid, *SD* standard deviation, *T1* dramatic tenor, *VE* vibrato extent, *VR* vibrato rate.

**Figure 1:**
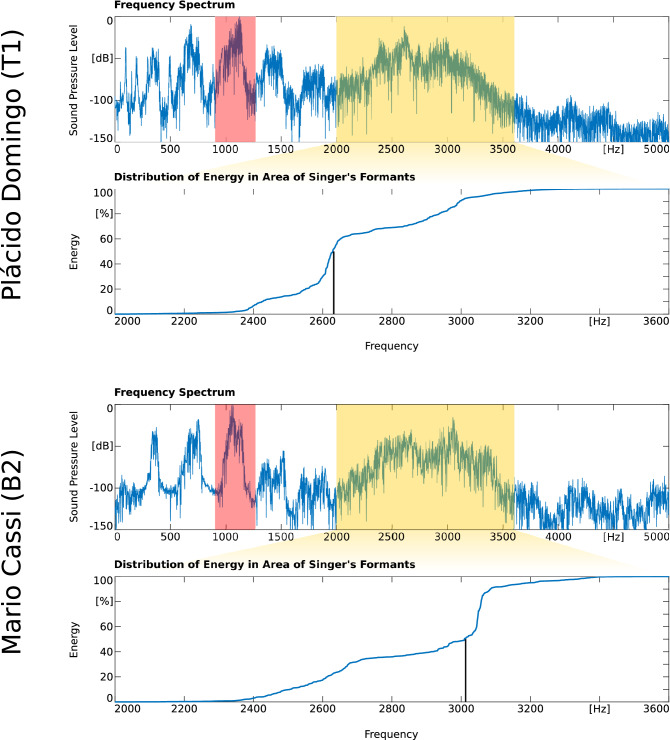
Comparative presentation of two FHE values for the dramatic tenor (T1) Plácido Domingo (2632 Hz) and the lyric baritone (B2) Mario Cassi (3013 Hz) at comparable pitch f4 (362 vs. 361 Hz). The yellow highlighted area marks the frequency band defined for the calculation of all timbre parameters. The vibrato is calculated at the harmonic marked in red (VR, VE). The lower half of both examples shows the progression of sound energy within the investigated frequency band. The black bar indicates the frequency at which 50% of the energy is reached (FHE).

In contrast to the group investigation, this comparison revealed a clearly lower FHE, PHE and SC value for the tenor than for the baritone. As expected, the selected sopranos showed a significantly lower FHE, PHE and SC value in the dramatic voices compared to the lyric voice. Additionally, the individual vibrato parameters VR and VE were considered for a more detailed analysis. Referring to recent investigations^19^, the following can be stated for the vibrato values: With Nilsson (S1), VE was clearly below the expected average and VR slightly below the 95% RR for dramatic sopranos. With Stemme (S1), VE was clearly above average and VR typical for dramatic sopranos. With Mazzucato (S2), VE was typical and VR slightly below the 95% RR for lyric sopranos. With Domingo (T1), VE was below and VR above the respective 95% RR for dramatic tenors. With Cassi (B2), VE was above and VR clearly below the respective 95% RR for lyric baritones. The parameters FHE, VR, and VE were highlighted in the table [bold], since they are easy to understand from a pedagogical point of view and therefore particularly well suited for communication and teaching in voice consultations. The great visual expressiveness of FHE becomes evident in Fig. 1, where Domingo and Cassi were compared. It demonstrates the individual progression of sound energy within the specified frequency band from 2000 to 3600 Hz. The black bar indicates where 50% of the energy was reached, representing the individual FHE value. The analyzed sound samples were taken from the role of Rodolfo from the opera Luisa Miller by Verdi (Domingo) and the role of Malatesta from the opera Don Pasquale by Donizetti (Cassi).

**Figure 2:**
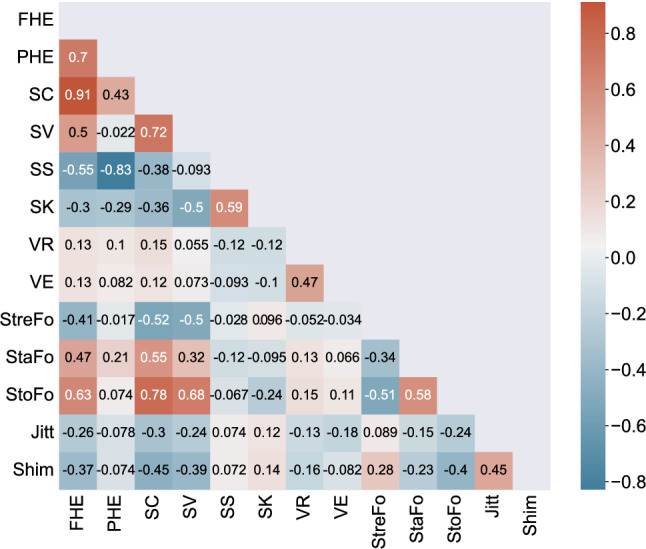
Correlation heat map showing the relationships between candidates for the input features in a random forest model. *FHE* frequency of half energy, *Jitt* Jitter, *PHE* position of half energy, *Shim* Shimmer, *StaFo* Start of formant, *StoFo* Stop of formant, *StreFo* Strength of formant, *SC* spectral centroid, *SK* spectral kurtosis, *SS* spectral skewness, *SV* spectral variance, *VE* vibrato extent, *VR* vibrato rate.

Figure 2 shows the correlation heat map between all investigated vocal parameters, which revealed high correlations between FHE, PHE, and SC. In ensemble learning, similar features used as input for the base learner can impair the robustness of the model^23^. Since the characteristics defined by FHE and PHE are practically the same (i.e. frequency or position of half energy), only one of both parameters needed to be retained. From Eq. (2) (cf. Materials and Methods) it can be deduced that the ratio of the SD to the mean of PHE is always greater than that of FHE. This greater ratio is a more beneficial property for classifiers to determine the hyperplane in the feature space. Compared to FHE, PHE also had a lower correlation with SC. Therefore, only PHE and SC were chosen to represent the energy distribution information of the samples throughout the machine learning process.

**Figure 3:**
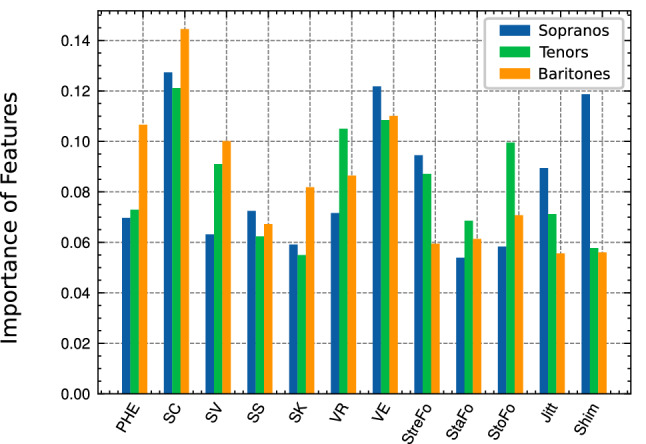
Importance of different features for voice structure classification of the investigated sopranos, tenors, and baritones (balanced error rates: 18.0%, 19.6%, and 17.5% respectively), based on information gain.

Taking various vocal parameters into account proved to be crucial for the most efficient automated voice structure classification. The importance of PHE and SC for the overall classification of the soprano, tenor and baritone voices is presented in Fig. 3. The bar chart visualizes the influence of all features used in the voice structure classification. For all examined voice types it was shown that the parameters presented here play an essential role in the objective description of the timbre. In addition to the vibrato parameters VE and VR, the timbre features PHE and SC also made an effective contribution to the classification, with SC having the greatest influence in all voice types.

**Figure 4:**
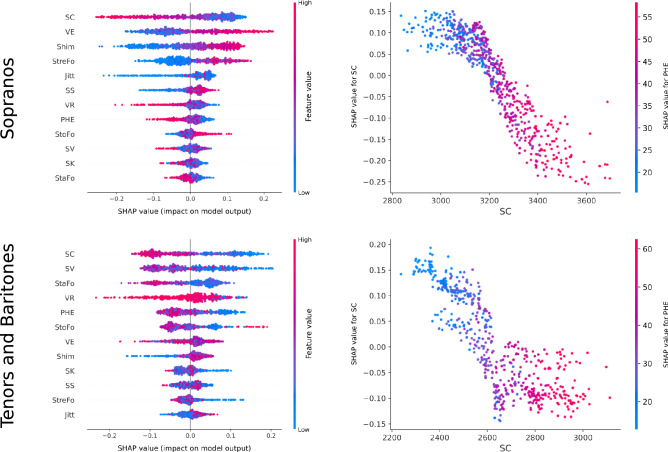
Left side: SHAP summary plots of input features for sopranos (upper row), as well as tenors and baritones (lower row). Features are sorted in descending order of the impact on the model output. Right side: SHAP dependence plots showing the impact of the interaction between SC and PHE. In all plots, only the results for the dramatic voice structure are shown.

Figure 4 gives a graphical interpretation of the classification model for sopranos, tenors, and baritones. On the left, the SHAP summary plots reveal that the SC had the greatest impact on the model output. As SC decreased, the likelihood that the model would classify a voice as dramatic increased, and vice versa. How the output of the model was impacted by the interaction of SC and PHE is shown on the right. A significant negative correlation was observed between the two features representing the energy information of formants and the predicted dramatic results. As with all binary classification problems, the SHAP summary plots were always symmetric for both classes, so only the impact of features on dramatic voices are presented.

## Discussion

The construction and first practical application of the new objective timbre parameters FHE, PHE, and SC were successful. Especially FHE, the calculated frequency at which 50% of the sound energy is reached within the specified frequency band, is very easy to understand and visualize. In order to describe the timbre even more precisely, SC was introduced as an additional feature, since it can be used very well to represent the energetic center of sound within the specified limits. However, since the mathematical description of this feature is much more complex compared to FHE, and thus the demands on voice teachers as "translators" between voice science and the "singer's world" increase significantly^14^, this study focuses on the FHE as a more comprehensible communication tool, because it is easier to explain and understand in pedagogical practice. The methodology presented in this paper can be used both to objectively verify the previous assumptions about timbre described in the introduction, as well as classify any sound samples in terms of voice type or voice structure (lyric vs. dramatic).

FHE means of the voice types in Table 1 confirm the distinction between a baritone- and tenor-like timbre^8^. Nevertheless, the generally assumed relative timbre difference of 200 Hz between 2 adjacent registers as presented in the literature turned out to be imprecise. According to our calculations, this difference is either notably below (251 Hz between tenor and baritone) or above (70 Hz between baritone and bass) the exact mean value. These findings represent a specification in the objective description of the absolute timbre characteristics in the investigated voice types. However, within the baritone voices, mean FHE difference between the lyric and dramatic voice structure was larger than 200 Hz, which suggests that lyric baritones could be assigned, at least in part, to the tenors. The timbre difference between both voice categories was also clearly recognizable in tenors (112 Hz) and sopranos (141 Hz). Apparently, previous reports about the SF center frequencies^9,24^ lack precision and completeness, since they focus on the assignment of voice types. In contrast, the newly introduced timbre parameters FHE, PHE and SC also reliably detect voice structure differences. Their use and implementation in voice diagnostics therefore expand the possibilities of a more specific evaluation of professional opera voices.

Contrary to all other voice types, the mean FHE difference within the basses is so small (8 Hz) that it can be considered meaningless. This supports previous findings of vibrato investigations in which basses formed an exception regarding voice structure classification^19^. The low frequencies needed for the dark timbre expected from basses on the opera stage prevent a clear differentiation between lyric and dramatic voices using the timbre parameters presented here. Interestingly, Kloiber also avoids the terms lyric and dramatic for basses despite employing them for all other voice types^12^. Only Ling^13^, in his attempt to reform the German “Fach” system, uses both terms also for the basses.

The interactions of voice types and voice structure (lyric or dramatic) can be easily understood by the presented comparison of the lyric baritone Cassi with the dramatic tenor Domingo. It also demonstrates the effectiveness of a combined analysis of timbre and vibrato parameters. Based on an approximately 300 Hz higher mean FHE value, it could be concluded that Cassi belongs to a higher voice type than Domingo. With his very bright timbre, Cassi could be assigned to the tenor voices. Domingo, on the other hand, showed timbre parameters just above the baritone average. With this dark timbre, “quite comparable to a lyric baritone”^25^, he could also be assigned to the baritone voice type. The additional consideration of vibrato values resulted in a more differentiated picture: Domingo's mean VR (6.26 Hz) and VE (74.05) are within the 95% RR of lyric tenors (6.17; 6.34 and 71.70; 78.89), but outside the confidence intervals of lyric or dramatic baritones^19^. This indicates that his voice is more likely belonging to a tenor. In Cassi, the low VR and high VE are typical findings for lyric baritones (within mean ± SD)^19^, despite his bright timbre. This confirms quantitatively and comprehensibly from the perspective of singing voice pedagogy that Cassi's assignment to the voice type lyric baritone is very likely correct.

The significance of such an objectively supported voice assessment is also reflected by statements of the German tenor Jonas Kaufmann. In his younger years, he "imitated a typically bright, light German tenor", but his voice "increasingly went on strike"^26^. When a teacher opened him the way to a darker sound color, he gradually identified with this new timbre and found the voice that has made him world-famous.

In the practice of singing voice pedagogy it is very important to perceive the sound quality as differentiated as possible. In addition to auditory training in musical education, voice teachers must be able to distinguish between different timbres in a very sophisticated manner. Perceiving the spectral structure of a sound is a necessary prerequisite for recognizing voice timbre independently from vowel formation. Computer programs such as VoceVista (Sygyt Software, Bochum, Germany) display the spectrum of the singing voice in real time and offer a good opportunity to train this ability specifically^27^. To compare the timbre within a voice type, sound samples characterized by high and low FHE values can be compiled for differentiation. Another instructive training is to evaluate a collection of sounds with the same or similar FHE values but different vowel colors. Overall, the new timbre parameters, especially the FHE, improve the possibilities for a differentiated description of resonance strategies as well as for the objective determination of voice types and voice structure.

## Conclusion and outlook

Once voice structure classification within the complex fach determination becomes an automated process, the objective features will play a key role in the evaluation procedure. As observed in initial investigations, a detection accuracy of approximately 80% can be considered very good, since the differentiation between the two classes lyric and dramatic is relatively small. This can be confirmed by singers whose voices slowly developed from lyric into dramatic over the course of their stage careers, as well as the existence of so-called “Zwischenfächer” (e.g. cavalier baritone)^12^. The classification of male voice types using the Random Forest classifier yielded a balanced error rate below 30%. These results are all very promising. In the future, the database will be extended and include sound samples from mezzo-sopranos to broaden the spectrum of voice types relevant to singing voice pedagogy. The authors hope that this will also provide answers to the exciting debate on classification of mezzo vs. soprano vs. “Zwischenfach”.

To the observer, the data analysis underlying the classification process and some of the applied features can seem like a blackbox due to their complexity. The specific influence and weighting of the individual features can neither be clearly determined by auditory perception nor in terms of singing voice pedagogy. This fact can be challenging for the trustful cooperation between engineers, phoniatricians and voice teachers. It is therefore all the more important to find objective, generally accepted characteristics which can be communicated comprehensibly in an interdisciplinary way. We believe that scientifically defined characteristics such as vibrato and timbre parameters fulfill that role and provide a valuable contribution to future voice diagnostics and fach determination. Our vision is based on the assumption that the automated digital voice classification will always be used as a helpful complement to the essential assessment of the experienced singing voice teacher.

## Supplementary Information

Supplementary Information.

## Data Availability

All data of this study is available in the Supplementary Data S1.
